# Surgical outcomes and risk factors for overall mortality in brain arteriovenous malformations patients: a retrospective analysis

**DOI:** 10.3389/fneur.2024.1428718

**Published:** 2024-08-22

**Authors:** Ioana Miron, Viorel M. Pruna, Dan M. Visarion, George E. D. Petrescu, Radu M. Gorgan

**Affiliations:** ^1^Department of Neurosurgery, “Carol Davila” University of Medicine and Pharmacy, Bucharest, Romania; ^2^Department of Neurosurgery, “Bagdasar-Arseni” Clinical Emergency Hospital, Bucharest, Romania

**Keywords:** arteriovenous malformations, prognostic factors, surgical treatment, long-term survival, interventional treatment, conservative treatment, eloquent AVMs, venous drainage

## Abstract

**Background:**

Brain arteriovenous malformations (AVMs) are challenging vascular lesions. Extensive follow-up studies are necessary to refine the therapeutic algorithm, and to improve long-term survival in these patients. The aim of the study was to assess surgical outcomes, and to evaluate overall long-term mortality in patients treated for brain AVMs.

**Methods:**

This retrospective single-center study included 191 patients with brain AVMs, admitted between 2012 and 2022. Clinical and angiographical particularities have been analyzed, to identify factors that might influence early outcome and overall long-term mortality.

**Results:**

Out of 79 patients undergoing surgery, 51 had ruptured AVMs with total resection achieved in 68 cases (86.1%). Deep venous drainage was associated with incomplete resection. Female sex, admission modified Rankin Scale (mRS) > 2, and eloquent location were independent predictors of poor outcomes. Multiple venous drainage was associated with a higher risk of worsened early outcome. Eloquent brain region involvement, conservative treatment, increasing age, admission mRS > 2, and comorbidities significantly decrease survival in brain AVM patients. Patients treated with interventional treatments had significantly better survival than the conservatively managed ones, when adjusting for age and admission mRS.

**Conclusion:**

The study identified female sex, poor neurologic status on admission and eloquence as independent prognostic factors for a negative outcome after surgery. Patients who received interventional treatment had significantly better survival than patients managed conservatively. We recommend employing tailored, proactive management strategies as they significantly enhance long-term survival in brain AVM patients.

## 1 Introduction

Brain arteriovenous malformations (AVMs) are challenging vascular lesions of great interest for neurosurgeons, facing many controversies regarding their therapeutic approach, especially in the post-ARUBA era (1–3). The estimated prevalence of incidental finding was 0.05% (4), while the annual risk of first hemorrhage was 1.3% in the “Multicentric AVM research study,” and 2.2% in another similar study by Gross and Du (5, 6). In patients younger than 50 years old, 27% of intracerebral hemorrhages (ICH) are secondary to a brain AVM (7) and given the persistent, life-time risk of cerebral hemorrhage, and the potential hazardous consequences of brain AVMs rupture, treatment strategy must be tailored according to many factors, like location, age, size, patient’s medical history, or hemorrhagic status.

The present study aims to analyze several prognostic factors that influenced the patient’s outcome and survival. Constantly assessing factors that might influence the patient’s outcome after the treatment can lead to a better stratification of the therapeutic algorithm. Kato et al. (8) published an expert consensus on the management of brain AVMs, stating the need for adding neurologic status on admission, among other characteristics, in a newer classification. We evaluated the decisive role of the patient’s neurologic condition at diagnosis in relation to the outcome and its significance in conjunction with other prognostic factors as well.

Moreover, to our knowledge, there are few long-term follow-up studies appreciating survival in patients harboring brain AVMs, and more data on this subject could bring some light into this great debate about the potential benefit of treating brain AVMs in comparison with a more conservative approach, regarding survival. We evaluated mortality rates in different categories of patients from our cohort, with a particular interest in patients treated with interventional therapy vs. conservative management, over a period of 12 years of follow-up.

## 2 Materials and methods

### 2.1 Study design

**Figure 1:**
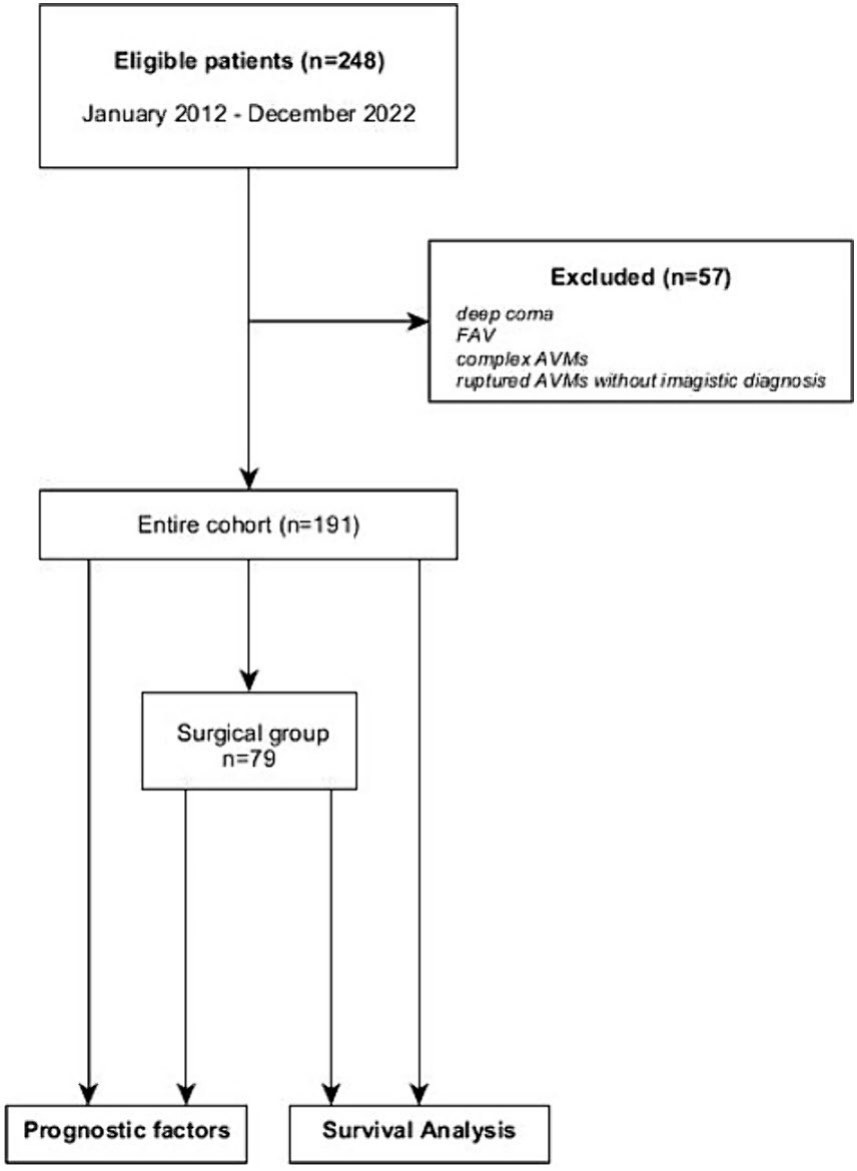
Flowchart depicting patient selection and aims of the study.

We conducted an observational single-center retrospective study at “Bagdasar-Arseni” Emergency Clinical Hospital. Inclusion criteria consisted of pediatric and adult patients diagnosed with brain AVMs who were admitted to our hospital between January 2012 and December 2022. All cases had an imagistic or histopathologic confirmation of the AVM. Patients who presented in deep coma (i.e., Glasgow Coma Scale—GCS 3 points with mydriasis, hemodynamically unstable), with ICH suggestive for AVM but whose clinical state did not permit further investigations were excluded. Arterio-venous fistulas (AVFs) have been excluded from the study, as well as complex malformations harboring an AVM with AVF or a brain AVM with extracranial AVMs. In 16 cases, the histopathologic diagnosis revealed brain AVM, but the only imagistic investigation available before surgery was a CT scan (ruptured AVMs). These cases were excluded due to the lack of morphologic characteristics necessary for accurate classification of the lesions, so the final cohort study had 191 patients (the flowchart of the study is represented in Figure 1). For data collection and reporting, we aimed to comply as thoroughly as possible with the recently published guidelines on reporting surgical and clinical outcomes for brain AVM studies (9).

### 2.2 Data collection

Demographic, clinical, and imagistic data were collected. The database included: sex, age, date of birth, Glasgow Coma Scale (GCS), modified Rankin score (mRS) on admission (adm-mRS), symptoms and neurologic deficits, Spetzler-Martin (SM) grade, Spetzler-Ponce (SP) grade and Supplemented Spetzler-Martin grade (SuppSM) for each AVM, rupture status on admission and during follow-up, presence of intraventricular hemorrhage (IVH), imagistic characteristics (location, eloquence, venous drainage pattern, number of arterial feeders, associated aneurysms, vascular steal), treatment modality, resection grade, complications, clinical outcome using mRS at discharge (dis-mRS), follow-up data, the presence of comorbidities. Additionally, SM grades were dichotomized into low to intermediate risk AVMs (SM grades I-III) and high risk (SM IV-V) AVMs. SuppSM grades were divided into three groups—low risk (SuppSM grades 2–4) lesions, those of intermediate risk (SuppSM grade 5 and 6) and high risk (SuppSM 7–10) AVMs.

Location was dichotomized in supra and infratentorial, but also noted separately in correspondence with the anatomical disposition (cerebral lobes, cerebellar, brainstem, ventricular, hemispheric, vermis, both supra and infratentorial). Eloquent locations included language, sensorimotor and visual cortex, brain stem, deep cerebellar nuclei, cerebellar peduncles, internal capsule, hypothalamus, and thalamus, as categorized by Spetzler et al. (10). Venous drainage was classified as superficial or deep (cases with mixed venous drainage were registered as deep). Venous ectasias, stenosis or intranidal fistulas were not noted. The number of arterial feeders was dichotomized between ≤2 and >2. Clinical outcome was considered favorable when dis-mRS ≤ 2. Aneurysms were registered as intranidal, flow-related, unrelated, or mixt (in cases harboring many types of aneurysms).

Imagistic diagnosis was achieved by digital subtraction angiography (DSA), MR angiography (MRA), CT angiography (CTA). Hemorrhagic status was noted in a binary manner at diagnosis as well as during the follow-up period. For the cases that ruptured during follow-up, date of event and mRS were also noted.

Treatment modalities included conservative (clinical and imagistic monitoring, anti-epileptic medication when needed), surgical resection, stereotactic radiosurgery (SRS), endovascular embolization [with ethylene-vinyl alcohol (EVOH)-based NALEAs (Onyx and Squid)], or a combination of the last three options. In multimodal therapeutic strategies, adjuvant treatment was noted separately. Surgical treatment was accomplished by several neurosurgeons from our institution. The endovascular treatment and the SRS procedures were both performed at the same institution. Treatment indications were established based on the clinical status of the patient, characteristics of the AVM, patient or caregiver option and the opinion of the medical team.

Follow-up data was extracted from the patient’s medical history. Surgical patients were followed at 2, 6, and 12 months postoperative. Afterwards, annually in the first 5 years, in cases with complete obliteration. For other therapeutic strategies, follow-up periods varied depending on the preference of the medical team and patient status.

Vital status at the end of the study (12th of January 2024) was obtained from the National Population Register Center.

### 2.3 Statistical analysis

Statistical analysis was performed using IBM SPSS Statistics for Windows, version 29 (IBM Crp., Armonk, N.Y., United States) and GraphPad Prism version 10.2.2 for Windows (GraphPad Software, San Diego, California United States). Pearson chi-square test and, when appropriate, Fisher exact test were used in univariate analysis to compare categorical variables. The Mann–Whitney U test was used to compare variables with non-normal distribution, like the SM and SuppSM grades. Statistical significance was considered at *p* < 0.05. Multivariate logistic regression analysis was used to evaluate independent predictors of outcome, introducing only variables that were significantly associated with the outcome in univariate analysis. Each multiple regression model had more than 10 participants per variable, as recommended by Hazra et al. (11). The area under the ROC curve was calculated for each multivariate regression model. The Log-rank test, as part of Kaplan–Meier survival analysis, was employed to assess the impact of several factors on mortality of all causes. Cox proportional hazard regression was used to evaluate independent risk factors for mortality in the entire cohort.

The present study was approved by the Ethics Committee of “Bagdasar-Arseni” Emergency Clinical Hospital (643/2024).

## 3 Results

### 3.1 General characteristic of brain AVM patients in the cohort

One hundred and ninety-one patients met the inclusion criteria, out of which 98 were male (51.3%). Patients’ age ranged between 5 and 79 years, with the median age being 36 years old. The median age of men in the entire cohort was 38.5, while the median age for women was 33, and this difference was significant in the Mann–Whitney test (*p* = 0.015). In the surgical group there were 40 men (median age 38) and 39 women (median age 34), with no statistically significant differences regarding the distribution of age between them. The mean follow-up time was 28 months (1–137 months). Four patients (2.1%) were lost to follow-up.

Ninety-four patients (49.2%) were diagnosed with ruptured AVMs, and 53 (27.7%) presented with IVH. Six patients suffered intracerebral hemorrhage (ICH) during the follow-up period, 5 cases being previously ruptured AVMs, and one case ruptured after 9 years of follow-up. Clinical characteristics are summarized in Table 1.

**Table 1:** Clinical characteristics of the patients included in the present study.

Variables	*n* (%)
Total	191 (100)
Male Sex	98 (51.3)
Age (years) - median (range)	36 (5–79)
Pediatric group	32 (16.8)
Headache	163 (85.3)
Seizures	78 (40.8)
Motor deficits	42 (22)
Cranial Nerve deficits	44 (23)
Comorbidities	42 (22)
Ruptured	
Yes	94 (49.2)
No	97 (50.8)
IVH	
Yes	53 (27.7)
No	138 (72.3)
GCS	
≥14p	154 (80.6)
9–13p	26 (13.6)
≤8p	11 (5.8)
adm-mRS score	
0	5 (2.6)
1	55 (28.8)
2	69 (36.1)
3	23 (12.1)
4	21 (11)
5	18 (9.4)

adm-mRS, mRS score on admission; IVH, Intraventricular hemorrhage; GCS, Glasgow Coma Scale.

Angiographic diagnosis (DSA) was available in 173 cases (90.6%), 3 patients underwent CTA (1.6%) and 15 cases underwent MRI with MR-angiography (MRA; 7.9%). The median SM grade was 3, and the median Lawton-Young (LY) grade was 3.

Small AVMs (nidus < 3 cm) were most frequent in our cohort (106 patients, 55.5%). Imagistic characteristics are summarized in Table 2.

**Table 2:** The imagistic characteristic of the 191 brain AVMs.

Variables	Total (%)
Total, *n* (%)	191 (100)
Localization, *n* (%)	
Supratentorial	169 (88.5)
Infratentorial	22 (11.5)
Deep	45 (23.6)
Spetzler-Martin Scale, *n* (%)	
Not determined^*^	1(0.5)
I	25 (13.1)
II	60 (31.4)
III	60 (31.4)
IV	30 (15.7)
V	15 (7.9)
Associated aneurisms, *n* (%)	
No	165 (86.4)
Nidal	13 (6.8)
Flow	12 (6.3)
Mixt	1 (0.5)
Diffuse nidus	20 (10.5)
Deep venous drainage	105 (55)
Unique venous drainage	114 (59.7)

^*^Venous drainage could not be characterized due to hematoma volume.

### 3.2 Therapeutic strategies

**Figure 2:**
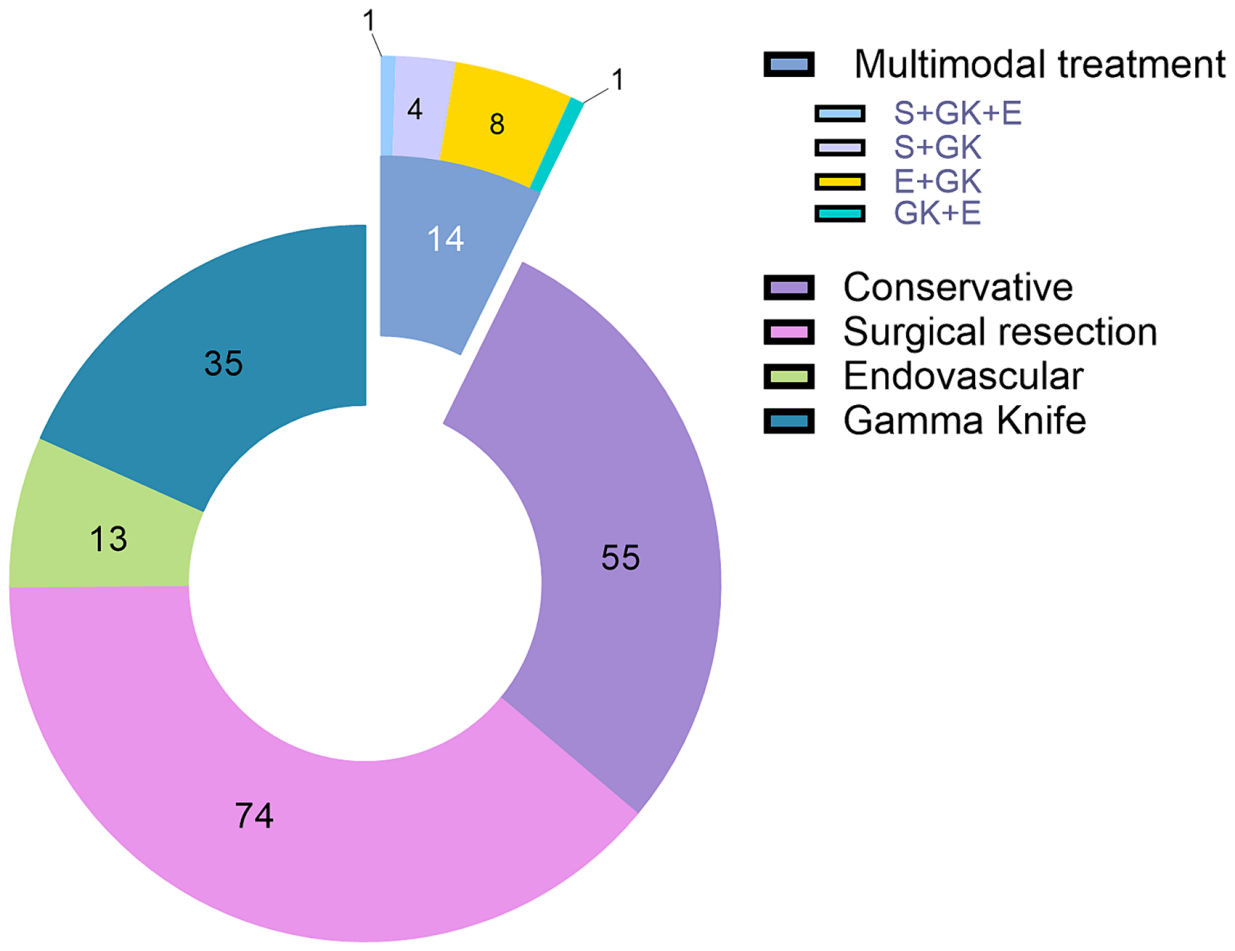
The distribution of patients in the cohort between different treatment selections.

The treatment of choice was microsurgical resection. Seventy-nine cases (41.4%) underwent surgery under general anesthesia. Five patients (6.32%) with ruptured AVMs needed emergency surgery. Twenty-one patients (11%) received endovascular treatment and 36 patients (18.8%) were treated with SRS. In 14 cases (7.32%) multimodal treatment was performed (the combined approaches are depicted in Figure 2). The conservatively managed group had 55 patients.

Of the 21 patients who underwent endovascular treatment as the first therapeutic options, 18 cases were unruptured AVMs. Thirteen AVMs were in eloquent location, while 14 presented deep venous drainage. Seven cases were low grade AMVs (3 SM I and 4 SM II lesions), 8 cases were SM III AVMs, and 6 cases were high grade AMVs (SM IV—5 cases and SM V—1 case). Median SM grade of the endovascular group was 3, and median SuppSM grade was 6. Two cases were infratentorial lesions, while five AVMs were classified as deep. Fifteen cases received one session, eight of them undergoing SRS in the following period, after the endovascular treatment. Six cases underwent two or three sessions of endovascular treatment (high grade AVMs).

#### 3.2.1 Surgical intervention and degree of resection

In 68 cases (86.1%) complete resection was accomplished after the microsurgical treatment; the median SM was 2 (range 1–5) and the median Supp-SM was 5 (range 2–8). The median SM grade of incompletely resected AVMs was 4 (range 1–5), and the median SuppSM was 5 (range 3–8). The SM grades of the brain AVMs differed significantly in the Mann–Whitney U test (*p* = 0.031) between the complete and incomplete resection groups. Of the 11 cases with incomplete resection, four were treated with SRS, and in one case embolisation and afterwards SRS were needed for complete obliteration. Two patients died during the same hospitalization, one with a ruptured SM IV AVM and the other with a ruptured SM V AVM who presented in poor clinical condition (adm-mRS 4). Four patients are currently under observation for small residual nidus.

We analyzed factors that influenced complete resection. Venous drainage location was available in 78 cases, equally distributed between deep and superficial. In 9 (23%) cases of the deep venous drainage group, complete resection could not be accomplished, and this characteristic proved to be significantly associated with incomplete resection (*p* = 0.047, OR 5.5, 95% CI 1.1–27.6). SP grade C AVMs were highly associated with incomplete resection; in comparison with SP A and B, 6 out of the 11 SPC lesions presented residual nidus after the microsurgical resection (*p* = 0.005, OR 6.1, 95% CI 1.5–23.6). No statistical difference was observed between SP A and B lesions.

#### 3.2.2 Interventional treatment vs. the conservative approach

The group of patients treated with any of the available treatment modalities (interventional treatment) and the conservatively managed group were compared to evaluate potential differences. Age at diagnosis (*p* = 0.418), and the presence of comorbidities (*p* = 0.971) did not differ significantly between groups, nor did the eloquence of the AVM (*p* = 0.142).

Median SM in the treated group was 2 (range 1–5), and median SuppSM was 5 (range 2–9). In the conservatively managed group, median SM was 3 (range 1–5), and median SuppSM was 6 (range 3–10). The Mann–Whitney U test was used to compare the differences in AVM grades between the two groups and proved that conservative management was associated with higher SM and SuppSM grades, *p* < 0.001.

### 3.3 Outcome and prognostic factors

The outcome was recorded using the mRS scale. One hundred and forty-eight (77.5%) patients were discharged in a good clinical and neurological state (dis-mRS ≤ 2). Worsened mRS was also noted (a negative difference between the adm-mRS and dis-mRS). 11 out of 191 patients (5.8%) were discharged with a worsened mRS.

#### 3.3.1 Prognostic factors in the surgically treated group

**Figure 3:**
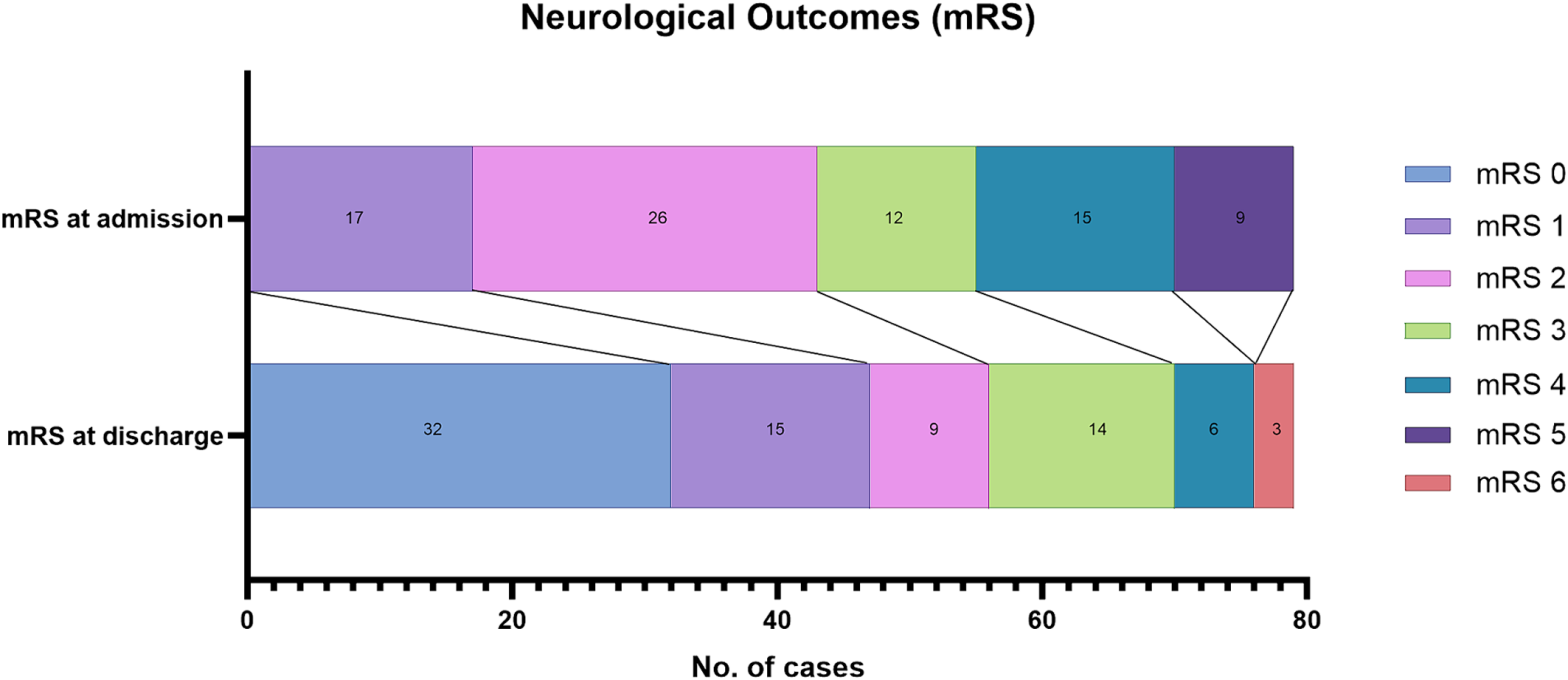
Neurologic status on admission and at discharge of the patients in the surgical group.

In the surgical group, 43 patients presented in good neurologic condition (adm-mRS ≤ 2), and 36 had adm-mRS values > 2. Fifty-five out of 79 patients (69.6%) were discharged with a favorable dis-mRS. In 65 cases (82.3%) the mRS improved, in 7 cases it remained unchanged, and only in 7 cases did the patient’s neurologic condition worsen (Figure 3).

We analyzed clinical and angiographic characteristics that could influence early outcomes. In the surgically treated group, following univariate analysis, female sex (*p* = 0.012), hemorrhagic presentation (*p* = 0.005), eloquence (*p* = 0.019), adm-mRS > 2 (*p* < 0.001), more than two arterial feeders (*p* = 0.008), more than one drainage vein (*p* = 0.043), deep location (*p* = 0.007) and intraventricular hemorrhage (IVH; *p* = 0.004) demonstrated to be risk factors for an unfavorable outcome, defined by dis-mRS > 2 (the univariate analysis including OR and 95% CI is depicted in Table 3). Due to the mild significance of the unique vs. multiple venous drainage impact on outcome, we ran the same analysis with 2 drainage veins as a cutoff, and results showed that AVMs with more than 2 drainage veins have significantly more chances of a negative postoperative outcome (*p* = 0.020).

**Table 3:** Univariate and multivariate logistic regression analysis—risk factors for poor outcome.

Risk factors (Surgery)	Univariate	*p*-value	Multivariate	*p*-value
	OR (95% CI)		OR (95% CI)	
Sex (Female)	3.64 (1.29–10.22)	0.012	3.32 (1.03–10.7)	0.044
Deep location	7.13 (1.66–30.70)	0.007		
>1 Drainage vein^*^	3.11 (1.01–9.55)	0.043		
>2 Drainage veins	3.23(1.17–8.9)	0.020		
>2 Arterial feeders^**^	3.93 (1.38–11.24)	0.008		
adm-mRS > 2	6.16 (2.09–18.19)	<0.001	4.68 (1.32–16.57)	0.017
IVH	4.23 (1.51–11.82)	0.004		
Eloquent area	3.66 (1.19–11.2)	0.019	3.79 (1.07–13.4)	0.038
Hemorrhagic presentation	5.88 (1.55–21.85)	0.005	2.79 (0.6–12.9)	0.189
Motor Deficit^***^	9.8 (3.2–29.8)	<0.001		
Aphasia^***^	4.1 (1.2–13.5)	0.016		

Risk factors (Entire cohort)	Univariate	*p*-value	Multivariate	*p*-value
	OR (95% CI)		OR (95% CI)	
Sex (Female)	2.09 (1.05–4.21)	0.036	3.01 (1.14–7.94)	0.025
Deep location	2.8 (1.34–5.85)	0.005	-	
adm-mRS > 2	24.13 (9.67–60.16)	<0.001	20.38 (6.53–63.55)	<0.001
IVH	5.18 (2.5–10.71)	<0.001	1.6 (0.57–4.73)	0.350
Eloquent area	6.11 (2.28–16.41)	<0.001	4.4 (1.37–14.1)	0.013
Hemorrhagic presentation	6.6 (2.86–15.22)	<0.001	1.6 (0.49–5.44)	0.426
Presence of aneurysms	2.48 (1.03–5.96)	0.038	3.2 (0.88–12.1)	0.075
Hydrocephalus^****^	15.07 (1.63–138.75)	0.01		
Motor deficit^***^	25.1 (10.8–62.9)	<0.001		
Aphasia^***^	7.2 (2.4–21.1)	<0.001		

^*^Angiographic description concerning the number of drainage veins was available in only 68 out of the 79 cases. ^**^Angiographic description concerning the number of arterial feeders was available in only 74 out of the 79 cases. ^***^Motor deficit and aphasia have not been used in multivariate analysis, being included in the calculation of mRS score on admission. ^****^Preoperative finding. IVH, intraventricular hemorrhage; OR, Odds Ratio; adm-mRS, mRS on admission.

**Figure 4:**
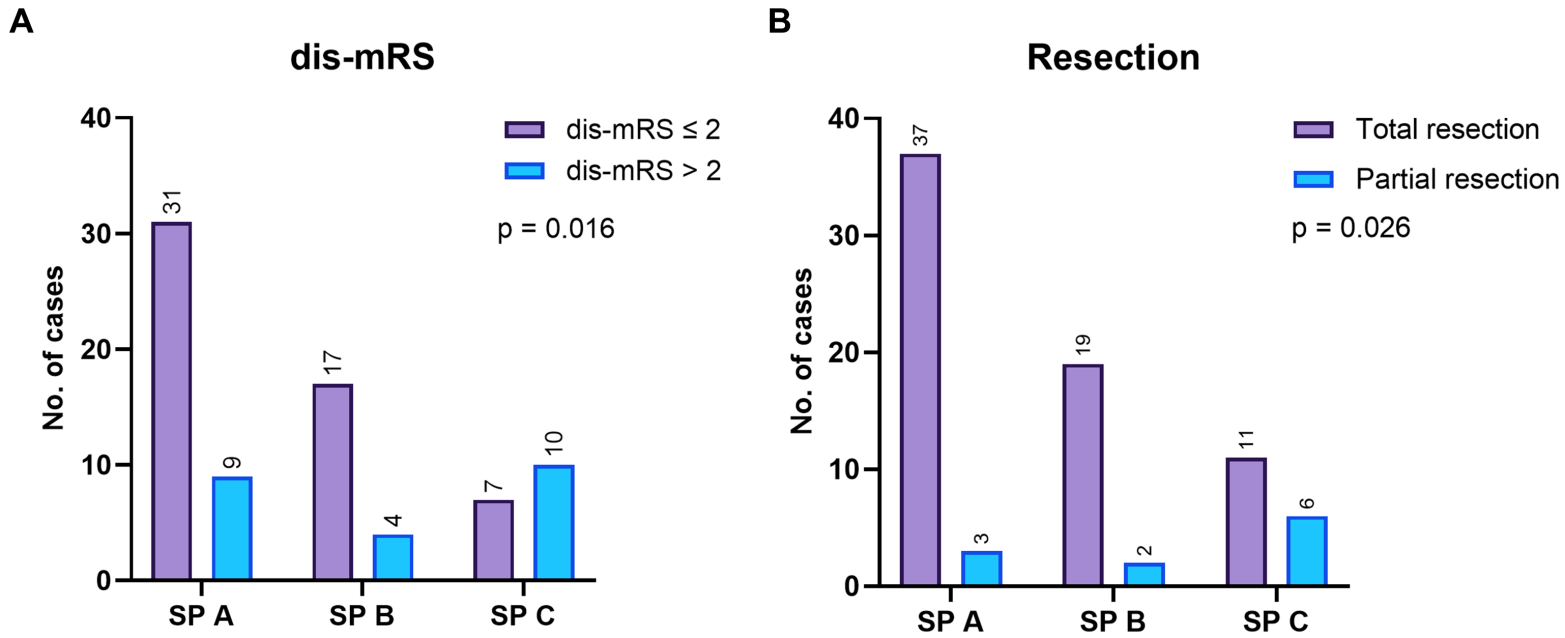
**(A)** The mRS at discharge (dis-mRS) distribution according to the Spetzler-Ponce (SP) grades, in the surgical group. **(B)** The resection grade achieved after surgery, according to the SP grades.

Univariate analysis demonstrated a significant correlation between the SP grade and an unfavorable postoperative outcome (Chi-square test, *p* = 0.016). There were 40 SP A AVMs, 21 SP B and 17 SP C lesions. The distribution of the postoperative outcome and the extent of resection according to the SP grading system is depicted in Figure 4. In terms of outcome, there were no significant differences between cases harboring SP A and B AVMs, but SP C AVMs had significantly higher chances of an unfavorable outcome.

High risks SM AVMs/SP grade C AVMs were significantly correlated with an unfavorable outcome (*p* = 0.003, OR 5.2, 95% CI 1.7–16.5). Ten out of the 23 (43.5%) operated patients who were discharged with mRS > 2 had high grade SM AVMs (SP C).

**Figure 5:**
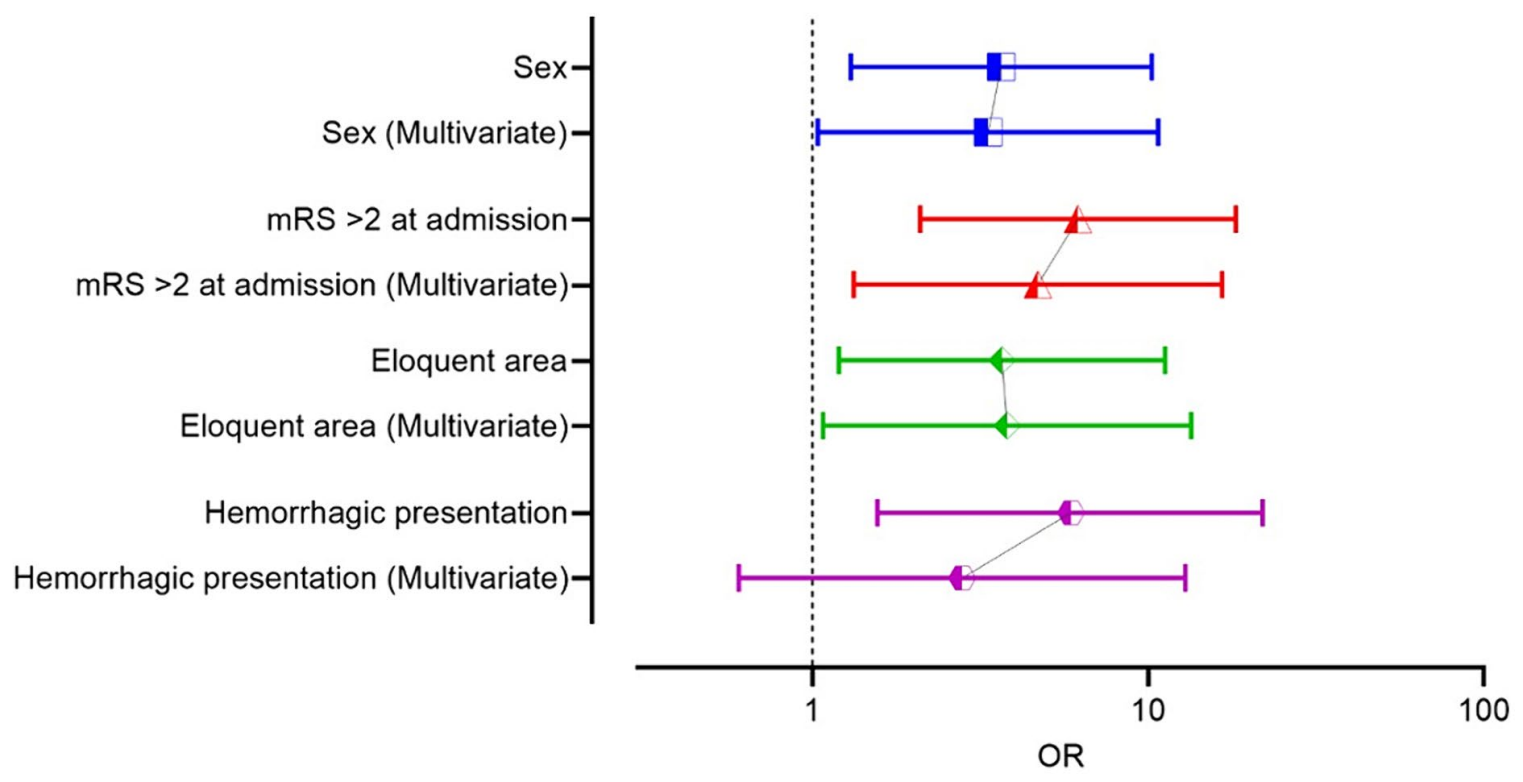
Comparative odds ratio (OR) of the risk factors in univariate and multivariate logistic regression in the surgical group.

**Figure 6:**
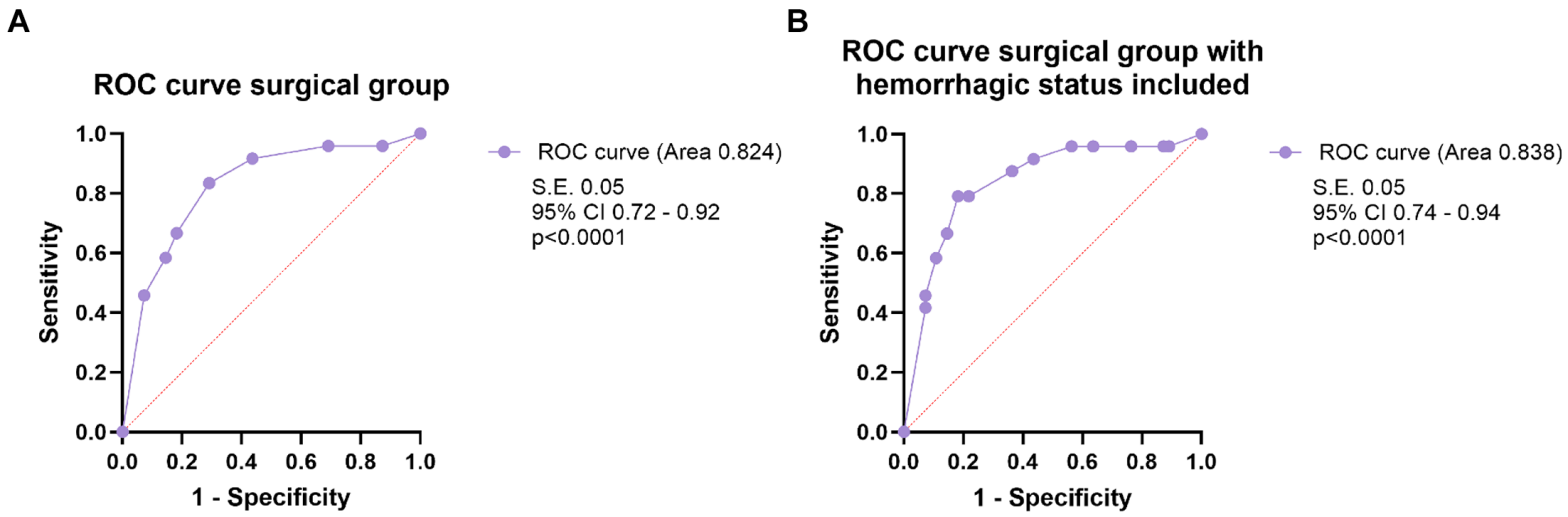
**(A)** The ROC curve analysis of the model including the three prognostic factors independently influencing the outcome (female sex, eloquence, mRS on admission >2), in multivariate logistic regression analysis, in the surgical group. **(B)** The ROC curve analysis of the model that includes the three prognostic factors independently influencing the outcome and the hemorrhagic status of the AVM, in the surgical group.

Multivariate logistic regression identified adm-mRS (*p* = 0.001, OR 6.7, 95% CI 2.1–21.6), female sex (*p* = 0.036, OR 3.45, 95% CI 1.1–11) and eloquence (*p* = 0.042, OR 3.6, 95% CI 1.05–12.4) as independent predictors of unfavorable outcome, although data should be interpreted cautiously due to confidence intervals of female sex and eloquence that barely exclude 1 (Figure 5). A receiver operating characteristic (ROC) curve analysis was conducted using the three independent risk factors determined through multivariate regression. The area under the ROC curve (AUROC) for the regression model was 0.824 (Figure 6). Deep location was not included in the model since 9 out of 10 deep surgically treated AVMs were scored as eloquent, nor the number of drainage veins and the number of arterial feeders, due to missing descriptions in some cases and the variable accuracy of different imaging techniques.

After adding hemorrhagic status to the regression model, all the other variables remained significant, and classification accuracy remained the same (78.5%), but hemorrhagic status did not retain its statistical significance, when controlling for the other variables (Table 3). Alternatively, variables were tested all together, and the model demonstrated the same independent variables: adm-mRS > 2, female sex and eloquence. We built, for comparison, a ROC curve with the full model, including hemorrhagic presentation that showed an area under the ROC curve of 0.838 (SE 0.051, *p* < 0.001, 95% CI 0.74–0.94).

It is important to mention that hemorrhagic presentation proved high statistical correlation with adm-mRS in univariate analysis, in the surgically treated group (*p* < 0.001, OR 15.2, 95% CI 4–57.6), and results were similar in the entire cohort as well.

Given this strong relationship between hemorrhagic presentation and adm-mRS, we analyzed the changes in mRS in the surgical group. Seven patients (8.9%) worsened after surgery, while the rest maintained the same neurological status or improved. Worsened outcome was associated with high-risk SM/SP C AVMs in univariate analysis (*p* = 0.037, OR 6, 95% CI 1.2–29.8). According to the SuppSM grading scale, there were 23 low-risk SuppSM AVMs, 40 of intermediate-risk and 15 lesions in the high-risk group. Of the 7 patients with worsened mRS, 1 (14.3%) was in the low-risk group, 2 (28.6%) were in the intermediate group, and 4 (57.1%) in the high-risk group. In univariate analysis, exact chi square test was significant (*p* = 0.045) showing a mild significant linear-by-linear association (*p* = 0.048) between increasing SuppSM grade and a worsened outcome. Furthermore, we observed a significant difference between brain AVMs with SuppSM higher than six, manifesting a much higher risk of worsening outcome in comparison with those below this cut-off value (*p* = 0.023, OR 7.3, 95% CI 1.4–37).

Multiple venous drainage demonstrated a tendency to increase the risk of worsened neurologic status after the surgery (*p* = 0.047, OR 6.2, 95% CI 1.04–37.6). The number of drainage veins was statistically correlated with the size of the AVM in our cohort (*p* = 0.009, OR 2.4, 95% CI 1.2–4.7).

#### 3.3.2 Outcome and prognostic factors in the entire study cohort

**Figure 7:**
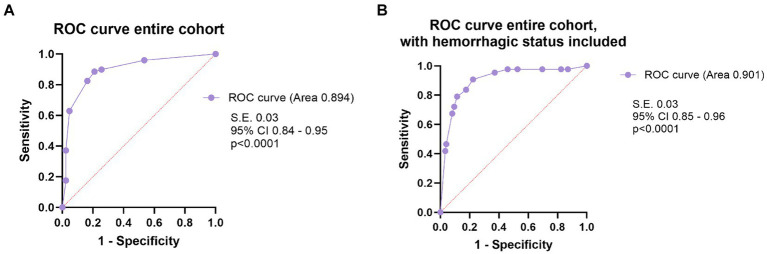
**(A)** The ROC curve analysis of the model including the three prognostic factors independently influencing the outcome (female sex, eloquence, mRS on admission >2), in multivariate logistic regression analysis, in the entire cohort. **(B)** The ROC curve analysis of the model that includes the three prognostic factors independently influencing the outcome and the hemorrhagic status of the AVM, in the entire cohort.

After analyzing the prognostic factors in the surgical group, we ran the same analysis using the entire cohort. Female patients had more often a negative outcome, compared to men (*p* = 0.036). Hemorrhagic presentation (*p* < 0.001), eloquence (*p* < 0.001), adm-mRS > 2 (*p* < 0.001), deep location (*p* = 0.005), IVH (*p* < 0.001) demonstrated significant correlation with the outcome in univariate analysis. Moreover, the presence of aneurysms was associated with an unfavorable outcome (*p* = 0.038). Similar to the surgical group, since almost all (42 out of 45) AVMs that were scored deep were eloquent, deep location was not introduced in multivariate analysis. All the risk factors that proved statistical correlation in univariate analysis were introduced in the logistic regression and only eloquence (*p* = 0.013), adm-mRS (*p* < 0.001) and female sex (*p* = 0.025) remained significant (Table 3). A ROC curve was then performed including the three significant variables, and the AUROC value was 0.894 (Figure 7). Following, we built a ROC curve with the whole model (including hemorrhagic presentation) that showed only a minimal increase in the predicted probability values (AUROC 0.901, SE 0.03, *p* < 0.001, 95% CI 0.85, 0.96).

Neurologic status on admission was relevant regarding the outcome even when only the unruptured lesions were analyzed. Nine patients with unruptured AVMs were admitted with mRS > 2 and 5 (55.6%) were discharged with similarly high values, mRS > 2. Eighty-five patients (96.6%) who presented in good neurological status maintained favorable mRS scores at discharge (*p* < 0.001). Other variables tested like deep venous drainage, age, location etc., did not demonstrate statistical correlation with the patient’s outcome in the unruptured AVMs cohort.

Of the 191 patients in our cohort, 180 improved or remained in the same neurological condition, and 11 worsened (5.76%). We analyzed what possible prognostic factors may lead to a worsening outcome (worsened mRS) and female sex and IVH proved mild statistical correlation in univariate analysis. Women were more likely to experience worsening of their neurologic condition, 9 (81.8%) of the 11 cases with a worsened outcome were females, while 96 (53.3%) patients from the group in which mRS remained unchanged or improved were males (*p* = 0.030, OR 5.1, 95% CI 1.1–24.4). Fifty-three patients presented IVH on admission, and 6 of them (11.3%) suffered a worsening of the neurologic condition (*p* = 0.041). On the contrary, of the 138 patients without intraventricular hemorrhage, only 5 (3.75%) were discharged with a worsened mRS. Regarding eloquence, although 10 out of the 11 cases in the worsened mRS were AVMs in eloquent areas, it did not reach statistical significance (*p* = 0.056) in univariate analysis. After introducing female sex and IVH in logistic regression, only the first prognostic factor remained borderline significant (*p* = 0.045, OR 5, 95% CI 1.04–23.8).

Rupture status was not statistically associated with a worsened outcome, neither in the surgical group (*p* = 0.694), nor in the entire cohort (*p* = 0.367), in univariate analysis.

### 3.4 Hemorrhagic and surgical morbidity

Following surgical treatment, in four cases (5.1%) the motor deficit worsened, and 3 patients (3.8%) presented with aphasia.

Five patients presented with hydrocephalus on admission, requiring surgery for shunting procedures. One case subsequently underwent surgery for AVM removal. Four out of five cases presented with ruptured AVMs with IVH and suffered secondary hydrocephalus due to IVH. Two of these patients died during the same hospitalization. Hydrocephalus (on admission) was a negative prognostic factor (*p* = 0.01), strongly related to IVH.

In 7 cases, multiple surgeries were needed during the same hospital stay. One patient operated for a ruptured posterior fossa AVM developed hydrocephalus, needing shunting. Four patients out of 79 (5.1%) suffered postoperative hematomas, requiring evacuation surgery, and this complication was significantly associated with an unfavorable outcome (*p* = 0.007, OR 1.2, 95% CI 1.003–1.435), but also with the worsening of the patient’s mRS score at discharge (*p* = 0.037, OR 14, 95% CI 1.6–121). All cases with postoperative hematomas were women and the correlation was borderline significant (*p* = 0.055). One case underwent emergency surgery to evacuate the hematoma, followed by DSA that confirmed the AVM, and a second surgery to resect the lesion. Two cases presented CSF leaks, with only one requiring a second surgery.

One patient had a late postoperative bone flap infection, 2 years after the surgery, requiring cranioplasty.

### 3.5 Survival analysis

Six patients (3.14%) died before being discharged, five of them being admitted in poor clinical condition (two patients were admitted mRS 4 and three patients severely impaired, mRS 5). Among these six patients, only three underwent surgery for AVM microsurgical resection.

Vital status was assessed on the 12th of January 2024, when out of the 191 patients in the study, 20 (10.47%) were deceased, half of them in the first 6 months, including the 6 patients who succumbed during the hospital stay, and 4 more in the following months (The number of deaths in different follow-up intervals is depicted in Table 4). In the surgical group, there were 7 deaths, 3 of them happening in the first 6 months. 10 patients were from the conservative group, one patient was treated with SRS, and in two cases patients had been treated with embolisation and SRS. The mean survival time was 131 months (S.E. 2.8, 95% CI 125–136).

**Table 4:** Mortality data by years of follow-up.

Time	Deaths
0–1 year	10^*^
1–5 years	6
5–10 years	4

^*^Died in the first 6 months after the diagnosis.

The median follow-up time for the survival analysis was 96 months (range, 1–144).

#### 3.5.1 Kaplan–Meier analysis

We performed Kaplan–Meier analysis to look for factors associated with overall mortality (of all causes) in brain AVMs in the surgical group and in the entire cohort. Median survival was not reached due to high censoring. The non-eloquent AVMs category had no events (all patients were alive by the end of the follow-up period). For higher accuracy, we reported survival at 6 months and at the end of the follow-up, along with mean survival, when available.

For the surgical group, increasing size (size <3 cm vs. size between 3 and 6 cm, *p* = 0.039; size <3 cm vs. size >6 cm, *p* = 0.007), eloquence (*p* = 0.021) and age > 40 (*p* = 0.028) were associated with increased mortality. High Spetzler-Ponce grades (SP C vs. SP A and B, *p* = 0.009), and high SuppSM grades (SuppSM > 6 vs. SuppSM < 4, *p* = 0.008; SuppSM > 6 vs. SuppSM 5–6, *p* = 0.039) were associated with increased mortality of all causes. Details about mean survival in months, survival at 6 months, and at the last follow-up in the surgical cohort are presented in Supplementary Table 1.

**Figure 8:**
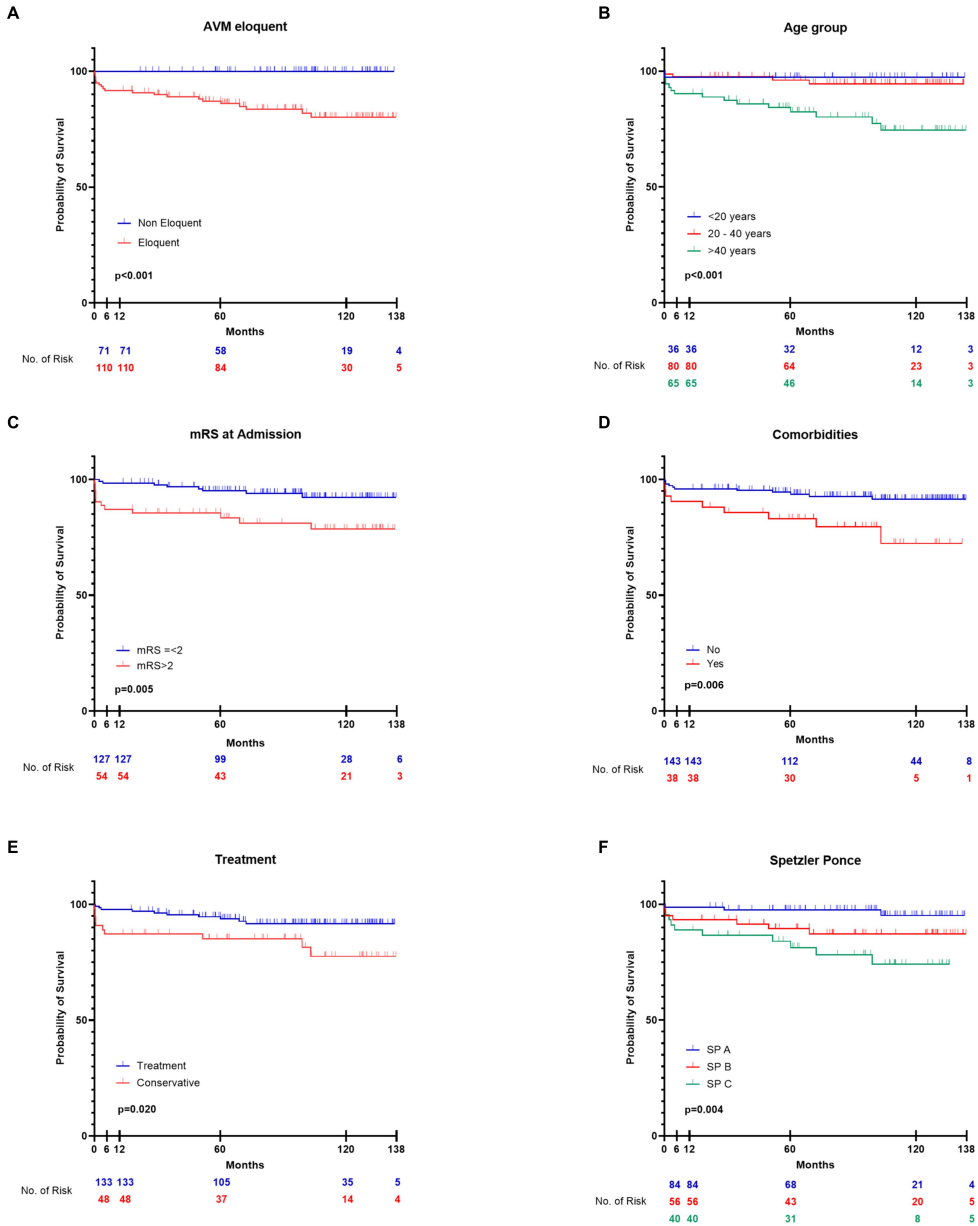
Comparison of survival rates according to several factors **(A–F)**, in the entire cohort, using Kaplan–Meier curves. **(A)** Survival patterns according to eloquence. **(B)** Survival of patients according to three age groups—patients younger than 20 years old, patients between 20 and 40 years old, and patients older than 40. **(C)** Survival of patients who presented in good clinical condition (mRS ≤ 2), vs. those who were neurologically impaired on admission (mRS > 2). **(D)** The impact of comorbidities in survival of patients with brain AVMs. **(E)** Survival of patients treated with interventional therapy (the treatment group), in contrast with patients managed conservatively (the conservative group). **(F)** Survival of patients according to the SP grade of the AVM.

When analyzing the entire cohort, we found that eloquence (*p* < 0.001), age according to LY classification age groups (patients < 20 vs. patients > 40, *p* = 0.01; patients aged 20–40 vs. those >40, *p* = 0.002), adm-mRS > 2 (*p* = 0.005), and comorbidities (*p* = 0.006) showed significant correlation with mortality (Figure 8). The 9 deceased patients with comorbidities were all older than 40 (median age 68), only 3 of them underwent surgery, and in two cases a partial resection was achieved. The patients in the treated group (surgical, endovascular, radiosurgery) had better survival than the conservative group (*p* = 0.020). High Spetzler-Ponce grades (SP B vs. SP A, *p* = 0.048; SP C vs. SP A, *p* < 0.001), and high SuppSM grades (SuppSM > 6 vs. SuppSM < 4, *p* = 0.001; SuppSM > 6 vs. SuppSM 5–6, *p* = 0.012) were associated with increased mortality. Details about mean survival in months, survival at 6 months, and at the last follow-up are presented in Supplementary Table 2.

Hemorrhagic presentation did not demonstrate a significant influence on mortality of all causes, neither in the surgical group (*p* = 0.786), nor in the entire cohort (*p* = 0.603), as determined by Kaplan–Meier survival analysis.

#### 3.5.2 Cox proportional hazards regression model

##### 3.5.2.1 Entire cohort

Given the low event rate (20 deaths in the entire cohort), we chose only the most relevant covariates for the Cox proportional hazards regression model to evaluate their impact on mortality of all causes, in the entire cohort. Patients from the conservative management group, as opposed to interventional treatment, were independently associated with a higher hazard of mortality (HR 2.7, 95% CI 1.1–6.6, *p* = 0.030). Poor neurologic status on admission (adm-mRS > 2) significantly increased the risk of mortality, when adjusting for other factors (HR 4.5, 95% CI 1.8–11, *p* = 0.001). Additionally, age > 40 was a significant predictor of mortality as well, with patients older than 40 experiencing a fivefold increase in risk compared to younger patients (HR 5.5, 95% CI 2–15.4, *p* = 0.001).

##### 3.5.2.2 SM I–III AVMs

Additionally, we assessed the differences in survival rates between the conservatively managed group and the treated (any treatment modality) group after excluding from the analysis high grade SM/SP C lesions. There were 145 patients, and 10 deaths. The conservatively managed group included 35 patients, while 110 patients received treatment. Patients from the conservatively managed group had a higher hazard of mortality (*p* = 0.010, HR 5.2, 95% CI 1.5–18) in comparison with the patients who received treatment, in univariate cox proportional hazard regression analysis. Upon adding age > 40 (*p* = 0.032, HR 4.5, 95% CI 1.1–17.8) and adm-mRS > 2 (*p* = 0.025, HR 4.3, 95% CI 1.2–15.4) to the model, the interventional vs. conservative approach variable retained its statistical significance (p = 0.010, HR 5.5, 95% CI 1.5–20). The patients that received any interventional treatment had significantly better survival than those in the conservatively managed group, even after excluding high grade SM/SP C AVMs.

## 4 Discussion

Patients harboring brain AVMs, if cured, may have promising long-term life expectancies. To date, few studies present extensive follow-up data on these patients. Our study addressed this gap of knowledge by providing an overview on long-term mortality, offering insight to refine the therapeutic algorithm and to improve long-term survival in these patients.

### 4.1 Surgical outcomes and prognostic factors

#### 4.1.1 Neurologic condition on admission

Stratifying surgical risks for brain AVMs has been of major interest for neurosurgeons, and many factors have been identified to influence the outcome after surgical treatment (12–21). In our logistic regression analysis, female sex, eloquence of the AVM, and poor neurologic status on admission proved to be negative prognostic factors in the surgical group, the most significant factor of all being the mRS score on admission > 2. Poor neurologic condition at presentation has been reported by many studies to influence the postoperative outcome in posterior fossa AVMs (15, 22, 23), but also in a series of Maalim et al. (17) regarding 169 patients surgically treated for brain AVMs. In a study on 224 patients operated for brain AVMs, Lawton et al. (13) studied how the hemorrhagic presentation of brain AVMs influences the postoperative outcome. Age, size, and eloquent location, along with mRS on admission, proved to independently influence the patient’s outcome, with individuals with ruptured AVMs presenting more often in poorer neurological condition, while those with unruptured lesions were almost intact or with minor symptoms.

Of all patients who underwent surgery in our study, 51 (64.6%) presented with hemorrhage, and 33 of them had adm-mRS scores >2, while only 3 unruptured AVMs were admitted with neurologic deficits. In univariate analysis there was a strong correlation between hemorrhagic status and the presenting neurologic condition (adm-mRS; *p* < 0.001, OR 15.2 95% CI 4–57.6), proving the strong relationship between the rupture of the AVM and the poor neurologic condition on admission. However, in multivariate analysis, adm-mRS was a stronger independent factor related to outcome compared to hemorrhagic status, and the latter did not retain its significance when adjusting for the other factors. Besides, we tested the relationship between adm-mRS and dis-mRS in the unruptured AVMs group, and the correlation was statistically significant in univariate analysis. These facts support the theory that symptomatic brain AVMs, no matter their hemorrhagic status, share the same risks (24).

When we analyzed the relationship between hemorrhagic presentation and a negative change in mRS (worsened mRS at discharge), no statistical correlation was found, neither in the surgical group, nor in the entire cohort. In Lawton’s study, unruptured AVMs were associated with neurological deterioration after the surgery, while those presenting with hemorrhage tended to improve after the surgery, also emphasizing the masking effect of hemorrhage on the surgical morbidity. This is especially true for AVMs in eloquent locations that present with hemorrhage, and with neurologic deficits due to the brain injury from hematoma expansion (13). The AUROC value of the multivariate regression model including mRS on admission > 2, eloquence and female sex demonstrated mild improvement after adding hemorrhagic status to the model (from 0.824 to 0.838), highlighting that although it does not independently impact the outcome, hemorrhagic presentation may have an indirect effect, by altering the mRS on admission. What we can conclude from these findings is that not the hemorrhagic presentation itself is independently affecting the outcome, but the clinical and neurological consequences produced by injuring the adjacent brain after AVM rupture. For this reason, we think that eloquent AVMs should be treated shortly after the diagnosis to prevent a future rupture. In unruptured eloquent AVMs, embolization or SRS should be considered, if possible, to prevent future morbidity associated with surgical intervention.

#### 4.1.2 AVM grading and risk stratification

Given the strong correlation between hemorrhagic presentation, neurologic condition on admission and the patient’s outcome, we analyzed the dynamic changes of the mRS values, and the factors that might lead to a potential worsening of the patient’s condition at discharge. Among the 79 patients who underwent surgery, seven experienced a deterioration in their neurologic status. SP grade C AVMs, as well as high grade SuppSM (SuppSM > 6) AVMs were statistically associated with a worsened mRS at discharge. These findings reinforce the fact that not all brain AVMs share the same risks, and that patients with SP grade A and B AVMs, or with SuppSM ≤ 6 grade lesions can and should be treated, to prevent a future hemorrhage and further morbidity. For high grade AVMs (SuppSM > 6), multimodal treatment is needed. If considered for surgery, preoperative embolization should be used, since it can lower the grade of the AVM.

#### 4.1.3 Venous drainage patterns

Our findings did not confirm a direct link between the size of the AVM and the risks of a worsened outcome after the surgery, as stated in other series (25), but we could notice that multiple venous drainage demonstrated a significant correlation with the risk of postoperative neurologic decline. Additional analysis revealed that the lesions with multiple venous drainage tend to be larger. Multiple venous drainage was associated with increasing size of the nidus in a series by Albert et al. (26), but also with higher SM grades and higher mRS scores on the long-term follow-up, in the pediatric series by Kellner et al. (27). Besides its classification as deep or superficial, the number of drainage veins might be another measurement of the AVM’s complexity, increasing the postoperative risk of impaired neurologic condition. Deep venous drainage (that also included mixt drainage in our cohort), increased the risk of incomplete resection. This conclusion might be important in deciding the most appropriate therapeutic option concerning AVMs with deep venous drainage, in cases where multiple treatment options seem appropriate.

Due to the heterogenous imagistic methods, we did not record data about venous aneurysms, ectasias or stenosis. Nevertheless, an increasing number of morphologic characteristics related to drainage veins are proving to be of significant relevance. The VALE score, developed by Chen et al., identifies venous aneurysms as the only protective factor for AVM hemorrhage, while deep location, exclusively deep venous drainage, and ventricular involvement were high risk factors for AVM rupture (28). We consider important to include all morphologic details about both feeding arteries and drainage veins in future studies, to identify new prognostic factors for brain AVMs.

#### 4.1.4 Outcome differences between male and female patients

Female sex was an independent risk factor for an unfavorable outcome in multivariate analysis, with women having a threefold increased risk of experiencing a negative outcome compared to men, in our cohort. Female sex was found to be a negative prognostic factor in other studies as well (12, 17). Although the mechanism is not readily explained, women are more prone to severe presentation and poor outcome in vascular surgery (29), as well as in patients with aneurysmal subarachnoid hemorrhage, probably because they are more prone to cerebral ischemia (30). After we analyzed the changes in mRS in the entire cohort, female sex was also associated with worsening of the neurologic condition at discharge, demonstrating a mild association in multivariate analysis as well. It was also associated with the occurrence of postoperative hematoma (all 4 cases of postoperative bleeding were in women). A possible explanation could have been increased age in women, but female patients were statistically younger than men in the entire cohort. Although we observed the same pattern in the surgical group, the difference did not reach statistical significance. Nevertheless, age was not an explanation for their less favorable outcome, but the theory of smaller, or more fragile arteries in women presented in vascular surgery (31) might be the reason for the association in our surgical cohort with a higher risk of postoperative bleeding, and with a less favorable outcome, in comparison with men. For these patients, preoperative embolization should be considered, especially in high-flow AVMs, to reduce the pressure and lower the chances of increased intraoperative bleeding and postoperative hematoma.

#### 4.1.5 Factors associated with a negative outcome in the entire cohort

Morgan et al. (3) stated the importance of analyzing risk factors not only in the surgical group, but also including an estimate of the unfavorable outcome in the non-surgical cohort. Therefore, to better understand our results, we analyzed prognostic factors in the entire cohort (surgical and non-surgical patients). Multivariate analysis proved that mRS on admission, eloquence and female sex were independent risk factors for a negative outcome in the entire cohort as well, and the strongest predictor of all was the neurologic condition on admission. Baharvahdat et al. (32) studied clinical outcome after embolisation of low-grade AVMs and poor neurologic condition before the intervention was the only independent predictive factor of poor outcome. These findings reinforce the need to constantly assess AVMs natural history and weight upon the consequences of a potential rupture, especially in an eloquent location, that can lead to severe neurological sequalae.

### 4.2 Survival analysis

Overall mortality in our cohort was 10.47%, 10 out of 20 deaths occurring during the first 6 months after the diagnosis. The first year after the diagnosis of an AVM is the period when most of the bleeding-associated deaths and treatment-associated mortality occur (33). According to Laakso et al. (33), mortality related to AVMs still represents half of all deaths within the first 10 to 20 years of follow-up, while in the 20-year follow-up study of Crawford et al., the overall mortality rate was 29, and 65% of were AVM-related deaths (34). Even though we did not have specific information regarding the mortality cause, given these findings, we decided to evaluate potential risk factors that might have affected overall survival in our cohort.

#### 4.2.1 Interventional vs. conservative treatment

Patients treated with interventional therapy (surgery, SRS, embolisation) had significantly better survival than those who received conservative treatment. Although the conservatively managed patients represented 28.8% of the entire cohort, half of all deaths were from this group. We acknowledge that this category comprises a heterogeneous population in previously published papers, including patients refusing any treatment, elderly patients or with comorbidities, and high grade AVMs for which treatment might have higher risks than the natural history. Criteria might differ from one cohort to another, and from one neurosurgical center to another. In our cohort, however, there were no significant differences regarding age, or the presence of comorbidities between the interventional and conservative groups, but the latter group had higher SM grades, so part of this difference in overall survival might be due to the intrinsic poorer prognostic of SM IV and V AVMs. For this reason, we excluded SM grade IV and V AVMs, and even in these conditions, patients who underwent interventional treatment demonstrated significantly lower risks of overall mortality, compared to patients managed conservatively. Our survival analysis reinforces the fact that, with careful selection, whenever the risk–benefit is adequate, brain AVMs, and especially SM grade I-III lesions, should be treated (3, 8, 35, 36).

Moreover, our comparison was between any interventional treatment (accounting also for the incomplete resection/obliteration cases, or multimodally treated ones) vs. the conservative approach and still survival in the first category was significantly better, in our 12-year follow-up study. Laakso et al., in their comprehensive long term follow-up study, found that complete AVM occlusion is most beneficial regarding survival, but also that partial treatment can improve survival in comparison with the conservative group, especially after 5–7 years of follow-up (33), although partial treatment is most often discouraged (37, 38). Further long-term follow-up studies are needed to better appreciate survival in these patients, in comparison with purely conservative treatment, especially after more than 5–7 years. Conservative treatment should remain the last therapeutic option, if the risk/benefit ratio for every therapeutic option is higher than the natural history of the AVM.

#### 4.2.2 Differences in survival based on eloquent location

Eloquence is a well-known prognostic factor concerning brain AVMs (18), and in survival analysis it proved to have high correlation with mortality. All deaths of all causes in our cohort were represented by patients with an eloquent AVM. Due to the 100% censoring in the non-eloquent group, we could not include eloquence in the Cox Proportional-Hazards regression model. However, eloquence might be an important factor regarding survival, and further studies are necessary to confirm the need for prioritizing treatment for this type of brain AVMs, to improve long term survival.

#### 4.2.3 Neurologic admission status and survival

In terms of survival, poor neurologic condition on admission increased overall mortality in the entire cohort, whereas hemorrhagic presentation did not. What we can stress out is that a brain AVM that becomes symptomatic and alters the patient’s mRS score may impact long term survival and require treatment, provided the risk/benefit ratio is reasonable. Furthermore, the decision-making process regarding patients with unruptured AVMs should consider not only the risks and benefits of elective surgery but also the risks of a conservative approach followed by surgery in case of hemorrhage, when an impaired neurologic condition may affect postoperative outcomes and overall survival.

#### 4.2.4 Age-related differences in survival

The age distribution, categorized into three according to the Supp-SM classification, aims to stratify population into pediatric (age < 20), young adults (age 20–40), and adults > 40 years old, category where we expect patients to present comorbidities (12). Hafez et al. (39) found age > 40 to significantly influence both early and late worse outcome after surgery. We performed a Kaplan Meier analysis to appreciate the impact of age on mortality of all causes in brain AVMs. In the surgical group, all patients younger than 20 were alive by the end of the study, so we dichotomized categories using 40 as a cutoff, and although the sample size was small, there was a statistical significance between groups, regarding survival. When analyzing the entire cohort, survival among patients aged >40 was found to differ significantly from the population < 20, as well as from young adults. Although we only have a perspective on deaths of all causes, and not specifically AVM-related ones, age > 40 impacts mortality in these patients. Given the fact that older age is also associated with feeding artery aneurysms (40) and venous ectasia, high-risk factors that are less frequent in children, necessitating time to develop (41), and that hemorrhagic risk in untreated AVMs is lifelong and increases with age (42), it is important to adequately counsel patients not only about surgical/procedural risks, but also regarding the negative impact of aging with a conservatively treated AVM, that might as well need therapeutic intervention at an older age.

#### 4.2.5 Independent risk factors for overall mortality

To better appreciate the influence of these factors on survival, we performed a Cox PH regression model that revealed that neurologic status on admission had a significant influence on long term survival, even after adjusting for the other risk factors. The analysis also demonstrated the impact of age on long-term survival and the potential benefits of interventional treatment over conservative management. It is important to mention that the findings of our survival analysis need to be interpreted cautiously, given the fact that we could only collect data regarding mortality of all causes.

## 5 Limitations

Our study has limitations. It is a retrospective, single-center, observational study, with inherent selection bias. The sample size is relatively small, reducing the power of the statistical analysis in some cases. We focused on prognostic factors for early outcomes because the study, by its retrospective nature, lacked standardized long term follow-up intervals, especially in the first years of the study. Moreover, since we could only obtain the vital status of the patients in our cohort, and not the cause of death, survival analysis depicts only data regarding overall mortality. A more standardized follow-up protocol with longer follow-up time and precise data over the cause of death is needed to better appreciate prognostic factors and survival rates in patients with brain AVMs.

## 6 Conclusion

The study identified female sex, poor neurologic status on admission and eloquence to independently influence the patient’s early outcome after the surgery. Furthermore, patients who received interventional treatment exhibited significantly better survival compared to those managed conservatively. We recommend treating brain AVMs, whenever the risk/benefit ratio is in the patient’s favor, to increase long term survival. Given the importance of the presenting neurologic condition on the postoperative outcome and on overall survival, treatment strategies regarding unruptured AVMs should be tailored after judicious counseling of the patient regarding the possible less favorable outcome after a future hemorrhage, and the cumulative age-related risks on survival.

## Data Availability

The raw data supporting the conclusions of this article will be made available by the authors upon request, without undue reservation.
