# Supplementary material for: Surgical outcomes and risk factors for overall mortality in brain arteriovenous malformations patients: a retrospective analysis
Source: Front Neurol. 2024 Aug 22;15:1428718. doi: 10.3389/fneur.2024.1428718 (PMC11374628; doi:10.3389/fneur.2024.1428718)
Supplement: Supplementary file 1 [file Data_Sheet_1.docx]

# Supplementary Figures and Tables

Table 1. Kaplan Meier Survival analysis in the surgical group

|  | **No. cases** | **No. of Deaths** | **Censored (%)** | **Mean survival in months**  **(95% CI)** | **Survival at 6 months (S.E.)** | **Survival at last follow-up (S.E.)** |
| --- | --- | --- | --- | --- | --- | --- |
| Eloquence |  |  |  |  |  |  |
| Non eloquent | 32 | 0 | 32 (100) | NA | 100% | 100% |
| Eloquent | 47 | 7 | 40 (85.1) | NA | 93.6% (0.036) | 83.3% (0.058) |
| Age group |  |  |  |  |  |  |
| < 40 years | 52 | 2 | 50 (96.2) | 138.6 (132,144) | 98.1% (0.019) | 95.7% (0.030) |
| ≥ 40 years | 27 | 5 | 22 (81.5) | 119,7 (102, 137) | 92.6% (0.050) | 80% (0.081) |
| AVM size |  |  |  |  |  |  |
| < 3 cm | 42 | 1 | 41 (97.6) | 140.2 (134,145) | 100% | 97.5% (0.025) |
| 3 – 6 cm | 34 | 5 | 29 (85.3) | 118 (104,131) | 94.1% (0.040) | 82.4% (0.073) |
| > 6 cm | 3 | 1 | 2 (66.7) | 90,7 (18,163) | 66.7% (0.272) | 66.7% (0.272) |
| Spetzler-Ponce |  |  |  |  |  |  |
| SP A | 40 | 1 | 39 (97.5) | 140 (134,145) | 100% | 97.4% (0.026) |
| SP B | 21 | 2 | 19 (90.5) | 132.5 (119,145) | 100% | 88.2% (0.078) |
| SP C | 17 | 4 | 13 (76.5) | 106.6 (81,131) | 82.4% (0.092) | 74.9% (0.110) |
| SuppSM |  |  |  |  |  |  |
| SuppSM Low risk | 23 | 0 | 23 (100) | NA | 100% | 100% |
| SuppSM Interm Risk | 40 | 3 | 37 (92.5) | NA | 100% | 91.2% (0.049) |
| SuppSM High risk | 15 | 4 | 11 (73.3) | NA | 80% (0.103) | 71.1% (0.124) |

Table 2. Kaplan Meier Survival Analysis in the entire cohort

|  | **No. cases** | **No. of Deaths** | **Censored (%)** | **Mean survival in months (95% CI)** | **Survival at 6 months (S.E.)** | **Survival at last Follow-up (S.E.)** |
| --- | --- | --- | --- | --- | --- | --- |
| Eloquence |  |  |  |  |  |  |
| Non eloquent | 71 | 0 | 71 (100) | NA | 100% | 100% |
| Eloquent | 120 | 20 | 100 (83.3) | NA | 91.7% (0.025) | 80.2% (0.042) |
| Age group |  |  |  |  |  |  |
| < 20 years | 37 | 1 | 36 (97.3) | 139.2 (131,146) | 97.3% (0.027) | 97.3% (0.027) |
| 20 - 40 years | 82 | 4 | 78 (95.1) | 136.8 (131, 142) | 97.6% (0.017) | 94.6% (0.027) |
| > 40 years | 72 | 15 | 57 (79.2) | 117.8 (l05,130) | 90.3% (0.035) | 74.6% (0.060) |
| adm-mRS |  |  |  |  |  |  |
| adm-mRS ≤ 2 | 129 | 8 | 121 (93.8) | 136.5 (131,141) | 98.4% (0.011) | 92.3% (0.028) |
| adm-mRS > 2 | 62 | 12 | 50 (80.6) | 118 (195,131) | 87.1% (0.043) | 78.6% (0.056) |
| Comorbidities |  |  |  |  |  |  |
| No | 149 | 11 | 138 (92.6) | 134.5 (129,139) | 96% (0.016) | 91.4% (0.026) |
| Yes | 42 | 9 | 33 (78.6) | 115.2 (99,130) | 90.5% (0.045) | 72.4% (0.091) |
| Treatment choice |  |  |  |  |  |  |
| Conservative | 55 | 10 | 45 (81.8) | 120.5 (107,133) | 87.3% (0.045) | 77.6% (0.068) |
| Treatment (all options) | 136 | 10 | 126 (92.6) | 134.3 (129,139) | 97.8% (0.013) | 91.7% (0.025) |
| Hydrocephalus on admission |  |  |  |  |  |  |
| No | 186 | 17 | 169 (90.9) | 132.4 (127,137) | 95.7% (0.015) | 89.4% (0.025) |
| Yes | 5 | 3 | 2 (40) | 73 (19,126) | 60% (0.219) | 40% (0.219) |
| Spetzler-Ponce |  |  |  |  |  |  |
| SP A | 85 | 3 | 82 (96.5) | 139.1 (134,143) | 98.8% (0.012) | 95.2% (0.029) |
| SP B | 60 | 7 | 53 (88.3) | 128.6 (118, 139) | 93.3% (0.032) | 87.2% (0.046) |
| SP C | 45 | 10 | 35 (77.8) | 110.6 (96,124) | 88.9% (0.047) | 74.1% (0.074) |
| SuppSM |  |  |  |  |  |  |
| SuppSM Low risk | 51 | 1 | 50 (98) | 140.3 (134,145) | 98% (0.019) | 98 (0.019) |
| SuppSM Interm Risk | 87 | 7 | 80 (92) | 133 (126,140) | 96.6 (0.020) | 89.9 (0.039) |
| SuppSM High risk | 52 | 12 | 40 (76.9) | 114 (100,129) | 88.5 (0.044) | 73.1 (0.069) |
